# AgriBioNER: A Named Entity Recognition Tool for Identification of ncRNA and Diseases in Agricultural Literature

**DOI:** 10.2174/0113892029435693260407094051

**Published:** 2026-04-15

**Authors:** Bhavesh Kumar Choubisa, Anu Sharma, K.K. Chaturvedi, Ragini Kushwaha, Mohammad Samir Farooqi, Dwijesh Chandra Mishra, S.B. Lal, Sudhir Srivastava, Amolkumar U. Solanke, Alka Arora, Girish Kumar Jha

**Affiliations:** 1 ICAR-IASRI, PUSA, New Delhi, 110012, India;; 2 ICAR-NIPB, PUSA, New Delhi, 110012, India

**Keywords:** Disease, spaCy, named entity recognition (NER), ncRNAs, NLP, plant genomics, large language model (LLM), transformer

## Abstract

**Objective:**

The rapid growth of agricultural and genomic research has generated a vast body of literature, making manual extraction of non-coding RNAs (ncRNAs) and agricultural disease entities increasingly challenging. Although pretrained transformer models such as BioBERT-v1.1, PubMedBERT-base-uncased-abstract-fulltext, DeBERTa-v3-large, and RoBERTa-base perform well in biomedical NLP, they lack task-specific Named Entity Recognition (NER) layers adapted to agricultural terminology. This study presents AgriBioNER, a domain-adapted NER framework for accurately extracting ncRNA and disease entities from agricultural literature.

**Methods:**

A curated dataset of agricultural abstracts related to ncRNAs and diseases was retrieved from PubMed. Four transformer models were fine-tuned using the spaCy transformer pipeline, and a classical spaCy model (en_core_web_lg) was trained separately. Performance was evaluated using precision, recall, F1-score, statistical significance testing, 500-iteration bootstrap validation, error analysis, and assessment of prediction time and scalability.

**Results:**

Transformer models showed consistent performance, while en_core_web_lg exhibited higher asymmetric errors. PubMedBERT-base-uncased-abstract-fulltext achieved the best results with a precision of 0.8351 ± 0.0139, recall of 0.8732 ± 0.0117, and F1-score of 0.8537 ± 0.0111. It processed abstracts in approximately 3.56 seconds per abstract, supporting large-scale deployment.

**Discussion:**

Domain-adapted transformer models substantially improved ncRNA–disease entity recognition in agricultural texts. Enhanced contextual representations enabled a better understanding of domain-specific terminology. These improvements demonstrate the effectiveness of specialized fine-tuning for agricultural NLP tasks.

**Conclusion:**

AgriBioNER offers a reliable framework for automated extraction of ncRNA and disease entities. The system reduces manual curation efforts while maintaining high accuracy and computational efficiency. It supports data-driven advancements in agricultural genomics and biotechnology research.

## 1 INTRODUCTION

The rapid growth of research has generated vast amounts of unstructured text. Finding useful information from this text is important for knowledge discovery. One emerging area of high relevance is the study of RNAs and their roles in various organisms. RNA plays a central role in the flow of genetic information from DNA to proteins, as outlined in the central dogma of molecular biology [1]. In this classic model, messenger RNA (mRNA) serves as the template for protein synthesis. However, advancements in transcriptomic technologies have revealed that a large portion of RNA molecules do not code for proteins [2, 3]. These transcripts are known as non-coding RNAs (ncRNAs). The ncRNAs, such as long non-coding RNAs (lncRNAs), small RNAs (sRNAs), miRNAs, circular RNAs (cirRNAs), and microRNAs, do not encode proteins. However, these ncRNAs play critical roles in regulating gene expression, growth and development, as well as in defence mechanisms against various diseases in organisms [4]. In the broader context of biomedical science, ncRNAs have been extensively studied for their roles in various diseases and immune responses [5, 6]. However, their importance is not limited to human health. In recent years, studies have shown that ncRNAs play a key role in regulating plant and livestock disease responses [7, 8]. LncRNAs and miRNAs regulate flowering, seed development, and stress tolerance in crops, and influence immunity, reproduction, and growth in animals [9]. These insights open new possibilities for crop and livestock improvement, making ncRNA research vital for sustainable agriculture and food security.

As ncRNA research expands, there is a growing need to identify disease-related ncRNAs in plants and animals by mining the vast agricultural scientific literature. However, extracting such information is challenging because scientific texts are dense, use inconsistent terminology, and lack standardized annotations, making manual curation slow and error-prone [10]. The challenge is even greater in agricultural science, where relevant information is fragmented across subfields and is far less curated than in the biomedical literature. To address the growing challenge of extracting biological information from rapidly expanding agricultural literature, Natural Language Processing (NLP) is becoming increasingly transformative. NLP systems can automatically analyze thousands of research papers to identify ncRNAs and diseases in crops and livestock. Although a range of text-mining techniques, including rule-based, dictionary-based, machine-learning, and deep-learning approaches, exists, they often struggle in specialized domains due to complex terminology, evolving vocabularies, and high contextual variability [11]. These challenges are especially pronounced in agricultural texts, where synonymy, abbreviations, and domain-specific expressions reduce the effectiveness of traditional NER methods. While pretrained biomedical NER models such as en_ner_bc5cdr_md [12], en_ner_bionlp13cg_md [13], and en_ner_jnlpba_md [14] perform well within their domains, they are not designed to jointly recognize ncRNA and disease entities and are trained on corpora that do not reflect agricultural contexts. Similarly, general pretrained language models like BioBERT-v1.1, PubMedBERT-base-uncased-abstract-fulltext, RoBERTa-base, and DeBERTa-v3-large [15-18] lack NER-specific classification heads and have not been fine-tuned for agricultural entity recognition, making their raw representations unsuitable for direct comparison. This creates a critical gap, as jointly extracting ncRNA and disease entities is essential for downstream tasks such as relation extraction, knowledge graph construction, and functional analysis, all of which are vital for advancing agricultural genomics, plant and livestock health, and crop improvement.

In this study, we created a custom ncRNA and disease NER dataset using relevant PubMed abstracts and fine-tuned several transformer-based models, including PubMedBERT-base-uncased-abstract-fulltext, DeBERTa-v3-large, RoBERTa-base, and BioBERT-v1.1, using SpaCy’s transformer pipeline (en_core_web_trf) [19]. These models were selected because they are fully compatible with the SpaCy transformer workflow. In parallel, the SpaCy (en_core_web_lg) [20] model was trained on the same dataset to serve as a non-transformer baseline. Based on comparative performance, PubMedBERT-base-uncased-abstract-fulltext achieved the highest precision, recall, and F1-score among all evaluated models. It was therefore selected as the underlying model for developing AgriBioNER. This user-friendly web-based tool is designed to extract ncRNA and disease entities from agricultural literature.

## 2 MATERIALS AND METHODS

The study followed several key steps to develop an NER system for agricultural literature. We collected, cleaned, and structured relevant scientific abstracts to build a dataset suitable for model training. Multiple models were then trained and fine-tuned to recognize domain-specific entities. Their performance was evaluated using precision, recall, and F1-score (reported as mean ± SD), and training progress was monitored to ensure proper convergence. We conducted comparative analyses to assess different models, including baseline approaches, and examined computational efficiency, prediction speed, and overall usability. Statistical significance was assessed, and qualitative analyses were performed to uncover common errors and patterns. Taking all evaluations into account, the best-performing model was chosen and deployed as a user-friendly web-based tool (Fig. **1**).

### 2.1 Dataset Preparation

#### 2.1.1 Data Collection

We collected abstracts on ncRNAs and diseases in the agricultural domain. We used an advanced query on PubMed, “Non-coding RNA AND Disease AND Agriculture NOT Human” (Fig. **2A**), which returned 2,652 (23 **‎**January **‎**2025) total abstracts published between 1988 and 2025 (Fig. **3**).

#### 2.1.2 Data Pre-processing

The raw data contains the institute name, the author’s name, DOI, journal name, *etc*., which are not required for our objective. We manually removed these entities from each abstract to ensure consistency and reduce noise (Fig. **2B**).

#### 2.1.3 Data Annotation Procedure

The corpus was manually annotated by two independent annotators to identify ncRNA and disease entities. A total of 2,652 PubMed abstracts were pre-processed and labeled using the NER Annotator (Fig. **S1**), a web-based tool compatible with the SpaCy pipeline [21]. All annotations were cross-checked by the lead annotator to ensure consistency and reliability. ncRNA mentions, including lncRNAs, miRNAs, siRNAs, piRNAs, snoRNAs, tasiRNAs, circRNAs, their full names, and gene symbols (*e.g*., miR397) were labeled as NON_CODING_RNA, while plant and livestock disease mentions (*e.g*., rice blast disease, bovine mastitis, tomato yellow leaf curl virus, bovine viral diarrhea virus) were labeled as DISEASE. Entity boundaries followed the maximum informative span rule such as complete biological names were fully annotated, punctuation was included only when part of standard nomenclature, ambiguous general terms (*e.g*., “pathogen infection”) were annotated only when referring to a specific disease, multi-word disease names and strain variants (*e.g*., BVDV-1) were treated as single entities, and nested entities were annotated at the highest meaningful level. To assess annotation quality, 50 sentences were independently annotated by both annotators, and disagreements were resolved through consensus, yielding a 98% match and a Cohen’s Kappa [22] score of 0.95, indicating an almost perfect level of consistency.

#### 2.1.4 Dataset Export

The annotated data were exported in a structured JSON format compatible with the SpaCy NER pipeline. The JSON schema shows the text and its entities along with their positions within the text. Each training instance consisted of a text string paired with a dictionary containing entity spans and labels (Fig. **2B**). Using this annotated corpus, a custom NER dataset (D) was constructed specifically to identify ncRNA and disease entities (Eq. 1).


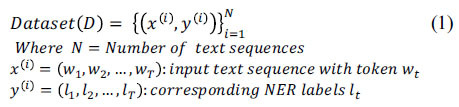


#### 2.1.5 Dataset Description

In the original dataset, there were 7,619 NON-CODING_RNA labels and 1,819 DISEASE labels, totaling 9,438 entity mentions. To address entity imbalance, we have applied Sentence-Level Resampling for NER [23] to increase the number of DISEASE labels, making them approximately equal to the number of NON-CODING_RNA labels. This process also led to a slight increase in NON-CODING_RNA entities, as many sentences containing DISEASE labels also included NON-CODING_RNA entities. After resampling, the dataset comprised 10,780 sentences, 8,142 DISEASE entities, and 9,782 NON-CODING_RNA entities.

The dataset was split into training, validation, and test sets at approximately 80:10:10 by sentence count. This resulted in 8,624 training sentences, 1,078 validation sentences, and 1,078 test sentences. Because entity mentions are unevenly distributed across sentences, the final label counts do not strictly follow this ratio. This is an expected outcome in sequence-labeling, token-level tasks such as BioNER. After splitting, the training set contained 6,498 DISEASE and 7,847 NON-CODING_RNA entities, the validation set included 829 DISEASE and 937 NON-CODING_RNA entities, and the test set contained 815 DISEASE and 998 NON-CODING_RNA entities (Table **1**).

### 2.2 Training and Fine-Tuning Models for NER

NER is a token-level classification task that identifies entities in a given text. Token-level classification assigns a specific label to every token in a sentence, enabling precise identification of entity boundaries. In this study, we fine-tuned (Eq. 2) several pre-trained transformer-based models for ncRNA and disease NER. These models included BioBERT-v1.1 from the DMIS Lab, a specialized biomedical adaptation of BERT; RoBERTa-base, a variant of BERT known for its optimized pretraining techniques; PubMedBERT-base-uncased-abstract-fulltext by Microsoft, pre-trained extensively on biomedical literature; and DeBERTa-v3-large, a general-domain model recognized for its language understanding abilities. Additionally, we trained a custom SpaCy (en_core_web_lg) model for domain-specific NER (Fig. **2C**).

The model used for NER is denoted as a function of 𝑓_𝜃_, where **𝜃** represents the learnable parameters of the model. Given an input of a sequence of tokens 𝑥(𝑖) = (𝑤_1_,𝑤_2_,...,𝑤_𝑇_), the model produces a corresponding predicted label sequence 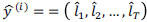. Here each 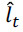 is the predicted named entity label for the token 𝑤_𝑡_. The model function 𝑓_𝜃_ is trained to learn this mapping based on the contextual representation of each token, enabling it to automatically assign appropriate entity labels in new, unseen data.


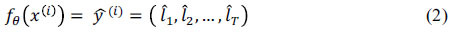


Where:


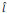
 : *predicted label*

𝑓_𝜃_ : *The modelfunction which maps input token to the output label prediction*

𝜃 : *parameters of the model*

### 2.3 Runtime Environment

The environment runs spaCy v3.8.5 on Python 3.11.12 on Linux (x86_64). All transformer models in this study were fine-tuned using the SpaCy transformer pipeline with en_core_web_trf (v3.8.0) to ensure consistent preprocessing, optimization, and evaluation. In contrast, the SpaCy (en_core_web_lg) model was trained using the standard (non-transformer) SpaCy pipeline (Table **2**).

### 2.4 Model Hyperparameters

We used the default hyperparameters provided by each model during both training and fine-tuning (Table **S1-S5**). This ensured consistency across all experiments and avoided model-specific tuning biases. All models were evaluated under the same standardized settings for fair comparison.

### 2.5 Model Evaluations and Comparisons

In this study, we conducted a comprehensive comparative evaluation of finetuned NER models on a test set. The goal was to assess their predictive robustness within an agricultural domain. All five models were evaluated using entity-level precision (Eq. 3), recall (Eq. 4), F1-score (Eq. 5), and Entity Error Percentage (Eq. 6). These metrics provided an assessment of each model’s ability to correctly identify entities. To establish the statistical validity of observed differences, we conducted pairwise McNemar tests [24]. Additionally, bootstrap resampling [25] with 500 iterations was performed to quantify the significance and variability of model predictions.

Beyond quantitative metrics, we performed comprehensive qualitative error analyses. These analyses identified systematic prediction errors, entity-specific misclassifications, and patterns in model behavior. Training dynamics were further assessed through loss curves and training progress visualizations, which provided insight into model convergence, overfitting, and stability during training. We also evaluated practical aspects of the models, including prediction speed, usability, and average prediction time per word, per sentence, and per abstract. These measures provided insight into the models’ efficiency and suitability for real-world applications, particularly for processing large volumes of agricultural literature. This combined evaluation strategy ensured a thorough understanding of the model's strengths, limitations, and practical utility for extracting meaningful biological and agricultural information from unstructured text.


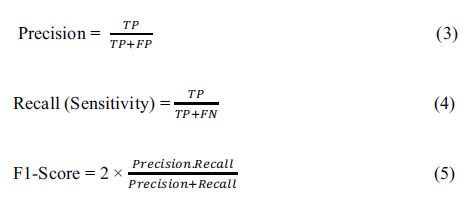



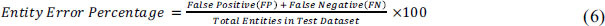


Where:

True Positives (TP): Model correctly predicted entities

False Positives (FP): The model predicted an entity that is not actually an entity.

False Negatives (FN): Model failed to identify a true entity.

Total Entities in Dataset: Total ground-truth entities in our labeled dataset.

### 2.6 Early Stopping and Model Selection

We used early stopping [26] based on the validation F1 Score to prevent overfitting during training. The F1-score was monitored across epochs, and the highest value was taken as the optimal checkpoint for each model. When the F1-score stopped improving, training was halted, and the best-performing version was automatically saved. After fine-tuning all models, their performance was compared using these optimal checkpoints.

### 2.7 Computational Setup, Cost, and Model Training Time

We used a one-time monthly subscription of Google Colab Pro+, which provided access to an NVIDIA A100 GPU [27] with 40 GB VRAM at a cost of USD 58.99. All experiments were conducted using this environment. Training duration varied across the evaluated models. SpaCy (en_core_web_lg) trained the fastest overall. Among the transformer architectures, PubMedBERT-base-uncased-abstract-fulltext achieved the shortest training time at 34.01 minutes. In contrast, BioBERT-v1.1, RoBERTa-base, and DeBERTa-v3-large required longer training durations, with DeBERTa-v3-large recording the highest training time at 59.00 minutes (Table **3**).

### 2.8 Web App Development

The web application was developed in Python and integrates several established libraries to support both backend processing and frontend visualization. The best-performing model identified during evaluation was employed for entity recognition (Fig. **2D**). NetworkX was used to construct entity-label networks, enabling the structured visualization of relationships between labels [28], while Matplotlib was used to render them. Flask served as the server-side framework, managing text input, file uploads, and processing workflows [29]. Bootstrap was used to design a responsive, user-friendly interface for end users [30]. Together, these tools ensured efficient entity extraction, clear network representation, high-quality visualization, and an intuitive overall user experience.

## 3 RESULTS

### 3.1 Training Progress of NER Models

Based on the comparative evaluation of precision, recall, and F1-score across all training steps, PubMedBERT-base-uncased-abstract-fulltext achieved the highest overall performance, ranking first with an F1-score of 0.8664 at step 3200 (Table **S6**). RoBERTa-base ranked second with an F1-score of 0.8619 at step 3400 (Table **S7**). BioBERT-v1.1, securing the third position with an F1-score of 0.8618 at step 5200 (Table **S8**). DeBERTa-v3-large achieved the fourth rank with an F1-score of 0.8590 (Table **S9**). Finally, the SpaCy (en_core_web_lg) model ranked fifth with an F1-score of 0.8351(Table **S10**). Overall, transformer-based models consistently outperformed the classical SpaCy (en_core_web_lg) model, and domain-specific pre-training (PubMedBERT-base-uncased-abstract-fulltext and BioBERT-v1.1) provided a measurable advantage in recognizing agricultural entities (Fig. **4**).

### 3.2 Training Loss Convergence Behavior of NER Models

The NER training loss for all models starts high at the beginning of training and gradually decreases over steps, showing effective optimization. Minor fluctuations occur due to batch-wise updates, but the overall downward trend indicates successful learning. PubMedBERT-base-uncased-abstract-fulltext (Table **S11**) and BioBERT-v1.1 (Table **S12**) exhibit higher initial losses, while DeBERTa-v3-large (Table **S13**), RoBERTa-base (Table **S14**), and SpaCy (en_core_web_lg) (Table **S15**) converge smoothly (Fig. **5**).

### 3.3 Validation Performance of NER Models

PubMedBERT-base-uncased-abstract-fulltext achieved the strongest validation performance with a precision of 0.8518, recall of 0.8814, and an F1-score of 0.8664. RoBERTa-base and BioBERT-v1.1 performed similarly, with RoBERTa-base reaching 0.8479 precision, 0.8763 recall, and 0.8619 F1, while BioBERT-v1.1 recorded 0.8337 precision, 0.8918 recall, and 0.8618 F1. DeBERTa-v3-large showed balanced performance with 0.8584 precision, 0.8595 recall, and 0.8590 F1. The SpaCy model (en_core_web_lg) ranked lowest, achieving 0.8300 precision, 0.8402 recall, and an F1-score of 0.8351 (Fig. **6**).

### 3.4 Model Performance on Test Dataset

Based on 500 bootstrap iterations, we observed noticeable differences in how the models behaved. PubMedBERT-base-uncased-abstract-fulltext emerged as the top-performing model with the highest F1-score (0.8537 ± 0.0111), just slightly ahead of BioBERT-v1.1, which achieved 0.8535 ± 0.0114. RoBERTa-base followed closely with an F1-score of 0.8493 ± 0.0113, showing strong overall performance. DeBERTa-v3-large, despite having high precision, showed more fluctuation in recall and therefore ranked lower with an F1-score of 0.8344 ± 0.0119. SpaCy (en_core_web_lg) had the lowest F1-score (0.8308 ± 0.0122) (Fig. **7**, Table **S16**).

### 3.5 Entity-Wise Performance

The entity-wise evaluation revealed clear differences in how the models handled the two biomedical categories. For DISEASE, PubMedBERT-base-uncased-abstract-fulltext achieved the highest F1-score (0.7261), followed closely by RoBERTa-base (0.7039) and BioBERT-v1.1 (0.7029). DeBERTa-v3-large achieved a slightly lower F1-score (0.6968), while SpaCy (en_core_web_lg) achieved the lowest DISEASE performance with an F1-score of 0.6042. In contrast, performance was substantially higher for the NON-CODING_RNA category across all models. PubMedBERT-base-uncased-abstract-fulltext achieved the highest F1-score (0.9004), with BioBERT-v1.1 (0.9002) and RoBERTa-base (0.9001) following extremely closely. DeBERTa-v3-large reached an F1-score of 0.8899, and SpaCy (en_core_web_lg) again trailed slightly with 0.8846 (Table **4, 5**).

### 3.6 McNemar and Bootstrap Test

The McNemar test showed no significant differences among transformer-to-transformer comparisons, indicating that these models made broadly similar types of errors despite small numerical performance differences. Only the comparison between PubMedBERT-base-uncased-abstract-fulltext and DeBERTa-v3-large showed borderline significance (*p* = 0.0502), but this was not supported by the bootstrap test (bootstrap *p* = 0.508), confirming that the observed difference was not statistically stable. In contrast, SpaCy (en_core_web_lg) consistently exhibited significant asymmetric errors when compared with BioBERT-v1.1 (McNemar *p* = 0.0010), PubMedBERT-base-uncased-abstract-fulltext (*p* = 0.0024), and RoBERTa-base (*p* = 0.0167). These findings were further supported by the bootstrap results, which showed higher variability and weaker agreement for SpaCy across pairwise comparisons, indicating that its predictions were less robust than those of the transformer models.

Within this landscape, PubMedBERT-base-uncased-abstract-fulltext emerged as the strongest overall performer. Although statistical tests did not reveal large differences among transformer models, PubMedBERT-base-uncased-abstract-fulltext consistently produced fewer unique errors and showed clear advantages over SpaCy (en_core_web_lg), while remaining highly competitive with BioBERT-v1.1, RoBERTa-base, and DeBERTa-v3-large. These combined McNemar and bootstrap results highlight the model’s stability and reliability for agricultural NER tasks (Table **S17**).

### 3.7 Quantitative Error Analysis

The error analysis reveals clear differences in model reliability across the five systems. BioBERT-v1.1 and PubMedBERT-base-uncased-abstract-fulltext show the strongest performance, with the lowest error rates (25.61% and 25.70%), indicating that they strike a good balance between false positives and false negatives and handle domain-specific entities more accurately. RoBERTa-base shows a slightly higher error rate (26.26%), while DeBERTa-v3-large performance declines further, driven mainly by a substantial rise in false negatives, which raises its overall error rate to 28.37%. SpaCy (en_core_web_lg) records the highest error percentage (28.90%) (Table **S18**).

### 3.8 Qualitative Error Analysis

All NER models showed three main types of mistakes, such as boundary or label confusion, false positives, and missed entities. Boundary and label confusion was the most common issue, in which the model correctly identified the entity but misaligned its span, either extending it, shortening it, or shifting its start position. False positives occurred when the model predicted entities that were not present in the gold annotations, often triggered by domain-specific keywords or repeated RNA mentions. Missed entities reflected difficulty in detecting less frequent or context-dependent terms, especially in complex sentences. Overall, most errors were structural rather than conceptual, suggesting the model generally understood the entity type but struggled with precise boundary detection (Table **S19**).

### 3.9 Baseline Model Performance on Our Domain-Specific Test Dataset

The baseline evaluation on our domain-specific test dataset shows that standard biomedical NER models exhibit suboptimal generalization to agricultural text. The model en_ner_bc5cdr_md, originally trained on the BC5CDR corpus for chemical and disease recognition, achieved very low performance: precision of 0.139, recall of 0.043, and an F1-score of 0.066, corresponding to an entity error rate of 96.6%. The other baseline models, en_ner_bionlp13cg_md, trained on the BioNLP13CG corpus for broad biomedical entities, and en_ner_jnlpba_md, trained on the JNLPBA corpus for genes and ncRNA, performed even worse, yielding zero precision, recall, and F1-score, with nearly all entities incorrectly predicted (100% error). These results clearly demonstrate that baseline biomedical NER models, despite being trained on high-quality biomedical corpora, fail to transfer effectively to our specialized domain (Table **S20**).

### 3.10 Usability, Scalability, and Efficiency of the finetuned PubMedBERT-base-uncased-abstract-fulltext Model

We evaluated the PubMedBERT-base-uncased-abstract-fulltext NER model on 339 agricultural abstracts, each averaging approximately 250 words. On average, the model processed a single abstract in about 3.56 seconds, corresponding to a throughput of roughly 202 words per second, 10 sentences per second, and 0.85 abstracts per second (Table **S21**). When processing multiple abstracts simultaneously, the model exhibited improved efficiency. For example, a single abstract took 1.77 seconds, whereas 100 abstracts were processed in just over 20.5 seconds, demonstrating strong scalability in batch mode (Table **S22**). For a substantially longer document containing approximately 167,000 characters, the model required around 222 seconds (Table **S23**), showing that while it can handle long-form text, processing time increases proportionally with document length. However, in a deployed web application, this processing time may increase further depending on platform capabilities, available computational resources, and concurrency limits.

### 3.11 AgriBioNER: A Web-Based Tool for ncRNA and Disease Named Entity Recognition

We developed AgriBioNER, a user-friendly, web-based tool for domain-specific ncRNA and disease NER from agricultural scientific literature. Implemented using HTML and Flask, the platform accepts both text file uploads and direct text input (Fig. **8**). Backend processing leverages the fine-tuned PubMedBERT-base-uncased-abstract-fulltext NER model, which achieved the highest F1-score among all evaluated models. Users can explore extracted entities through interactive tables, entity-label networks, and word clouds that visualize frequency patterns. The front-end is optimized for responsive, real-time interaction, enabling agricultural researchers to quickly analyze and interpret domain-specific information without programming expertise. Overall, AgriBioNER translates the predictive capability of transformer-based NER into an intuitive, practical tool for agricultural research. The web application is publicly accessible at: https://huggingface.co/spaces/BhaveshKChoubisa/AgriBioNER.

AgriBioNER generates five types of outputs that facilitate detailed exploration and analysis of the extracted entities. First, highlighted entity visualization (Fig. **9A**) allows users to see all identified ncRNA and disease mentions directly within the original text, making it easy to understand their contextual relevance. Next, a structured entity-label table (Fig. **9B**) lists each recognized entity along with its classification as either ncRNA or disease, and a word cloud visualizes the most frequently occurring entities. It helps users identify dominant themes in the input text (Fig. **9C**). In addition, AgriBioNER offers two interactive entity-label networks for ncRNAs (Fig. **9D**) and diseases (Fig. **9E**).

AgriBioNER provides an accessible and effective solution for biomedical text mining in agricultural research by integrating a high-performing domain-specific model with rich, interactive visualizations. It is especially useful for researchers working in genomics, plant pathology, and molecular biology, offering accurate and insightful extraction of ncRNA and disease-related information from scientific literature.

## 4 DISCUSSION

The rapid expansion of agricultural and plant biotechnology research has generated a vast, growing body of scientific literature, making manual extraction of relevant biological information increasingly impractical. This challenge motivated the development of AgriBioNER, a domain-specific system designed to extract ncRNA and disease entities from agricultural research articles. To support this goal, we conducted a comprehensive evaluation of transformer-based and classical NER models. Our aim was to determine the most accurate, stable, and deployment-ready architecture for agricultural biomedical text. Across training and validation experiments, transformer architectures consistently outperformed the SpaCy (en_core_web_lg) model [20]. They demonstrated a superior ability to capture complex contextual cues. Similarly, additional biomedical SpaCy baseline models, en_ner_bc5cdr_md [12], en_ner_bionlp13cg_md [13], and en_ner_jnlpba_md [14], failed to achieve competitive performance, showing noticeably lower precision, recall, and F1 Values across both ncRNA and disease entities. In contrast, transformer models, particularly PubMedBERT-base-uncased-abstract-fulltext [16], BioBERT-v1.1 [15], and RoBERTa-base [17], leveraged contextualized embeddings and domain-informed pretraining to deliver substantially stronger results. PubMedBERT-base-uncased-abstract-fulltext achieved the highest F1-score, followed closely by RoBERTa-base and BioBERT-v1.1 DeBERTa-v3-large [18] demonstrated moderate performance, while SpaCy (en_core_web_lg) lagged. These trends were also reflected in the test results derived from 500 bootstrap iterations [25]. PubMedBERT-base-uncased-abstract-fulltext achieved an F1-score of 0.8537 ± 0.0111, and BioBERT-v1.1 achieved 0.8535 ± 0.0114, making them the top performers. RoBERTa-base also performed strongly with an F1-score of 0.8493 ± 0.0113. In contrast, DeBERTa-v3-large showed lower and less stable recall, and the SpaCy model (en_core_web_lg) yielded the lowest F1-score of 0.8308 ± 0.0122. These results reinforce the conclusion that baseline models were ineffective for specialized agricultural NER, whereas transformer models delivered stronger, more reliable results.

Entity-wise analysis provided deeper insights into category-specific challenges. All models performed substantially better on NON-CODING_RNA, where contextual patterns were clearer, and the entity frequency was higher. PubMedBERT-base-uncased-abstract-fulltext, BioBERT-v1.1, and RoBERTa-base achieved nearly identical F1-scores of around 0.900, while DeBERTa-v3-large and SpaCy(en_core_web_lg) showed slightly lower performance. In contrast, DISEASE entities proved more challenging due to sparse mentions and heterogeneous phrasing. PubMedBERT-base-uncased-abstract-fulltext achieved the highest DISEASE F1-score of 0.7261, followed closely by RoBERTa-base at 0.7039 and BioBERT-v1.1 at 0.7029, indicating competitive performance among transformer models. DeBERTa-v3-large showed a moderately lower F1-score of 0.6968, while SpaCy(en_core_web_lg) recorded the lowest score of 0.6042, largely due to reduced recall. These findings highlight the importance of domain-specific pretraining for handling linguistically diverse biomedical entities and suggest that models trained on general or less specialized corpora struggle with contextually complex biomedical terminology.

Statistical significance testing further clarified model differences. McNemar’s test indicated no significant differences among most transformer models, confirming that their errors were highly overlapping. Only the comparison between PubMedBERT-base-uncased-abstract-fulltext and DeBERTa-v3-large approached significance, reflecting DeBERTa-v3-large’s unstable recall patterns. SpaCy (en_core_web_lg) differed significantly from all transformer models and produced a higher proportion of asymmetric errors, misclassifying entities correctly identified by transformers. Error analysis revealed that boundary inconsistencies, ambiguous keyword-triggered false positives, and missed multi-span entities were the most frequent errors across models. PubMedBERT-base-uncased-abstract-fulltext and BioBERT-v1.1 displayed fewer boundary and recall errors than RoBERTa-base or DeBERTa-v3-large. SpaCy (en_core_web_lg) produced more recall errors.

Taken together, these results underscore the clear superiority of domain-adapted transformer models for agricultural NER and highlight the limitations of baseline models. Given its excellent performance, stable recall, low asymmetric error rate, and efficient batch-processing inference, the PubMedBERT-base-uncased-abstract-fulltext model was selected for the AgriBioNER web application. AgriBioNER provides interactive visual outputs, including entity tables, entity-label networks, and word clouds, to support intuitive exploration of knowledge embedded in the scientific literature. Looking ahead, there are several ways to further enhance AgriBioNER. Improving the system to handle longer abstracts and full-text articles more efficiently could broaden its applicability. Expanding the annotated dataset to include underrepresented crops, disease subtypes, and additional entity types such as genes, drugs, and chemicals would help the model generalize better across diverse agricultural scenarios. Adding relation extraction and event detection capabilities could transform AgriBioNER from a tool that identifies entities into a platform capable of building comprehensive knowledge graphs for agricultural genomics. Together, these enhancements would make AgriBioNER more versatile and promote wider adoption of NER-driven insights in agricultural research.

## 5 STUDY LIMITATIONS

AgriBioNER is currently optimized for agricultural research literature, particularly for ncRNA and disease entities, and its performance may decline when applied to unrelated agricultural subfields or general biomedical texts. Newly discovered entities that were absent from the training data may be missed or mislabeled, and the system does not yet perform relation extraction or document-level co-occurrence analysis. Additionally, the underlying models may carry biases from their original biomedical training corpora, which can influence predictions in the agricultural domain. The comparison was limited to models compatible with SpaCy pipelines, thereby excluding several other transformer-based architectures. Additionally, processing very large corpora may demand substantial computational resources, posing practical challenges for large-scale deployment.

## CONCLUSION

In conclusion, AgriBioNER demonstrates that domain-adapted transformer models, particularly PubMedBERT-base-uncased-abstract-fulltext, offer superior accuracy, robustness, and scalability for extracting ncRNA and disease entities from agricultural literature. Transformer-based approaches effectively handled context-rich and imbalanced datasets, outperforming classical methods across both entity types. The system’s deployment facilitates intuitive exploration of literature through interactive visualizations and knowledge extraction. Future enhancements, including expanded datasets, model architecture, relation extraction, and edge-deployable models, will further strengthen its utility in agricultural research. Overall, AgriBioNER provides a reliable and scalable solution for automated text mining in agriculture.

## Figures and Tables

**Fig. (1):**
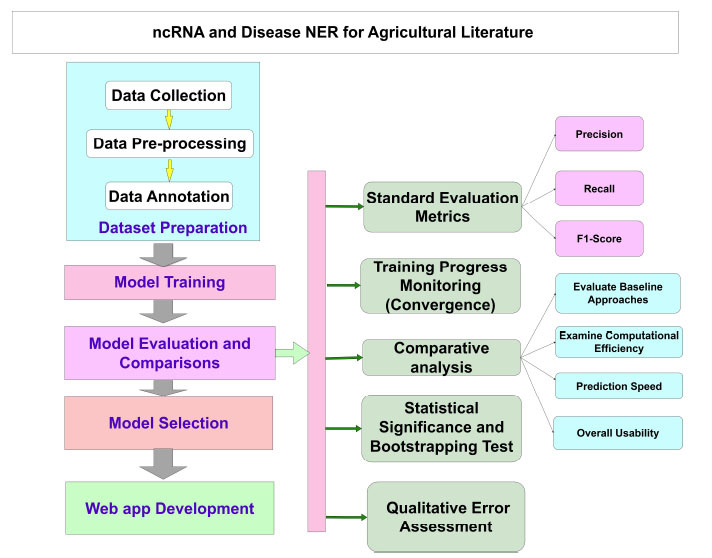
General workflow of the study.

**Fig. (2):**
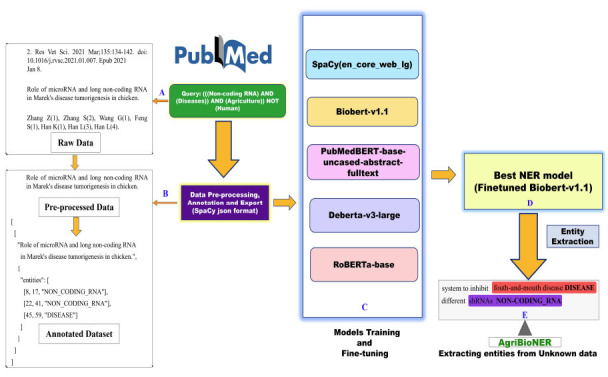
Workflow of the NER system development.

**Fig. (3):**
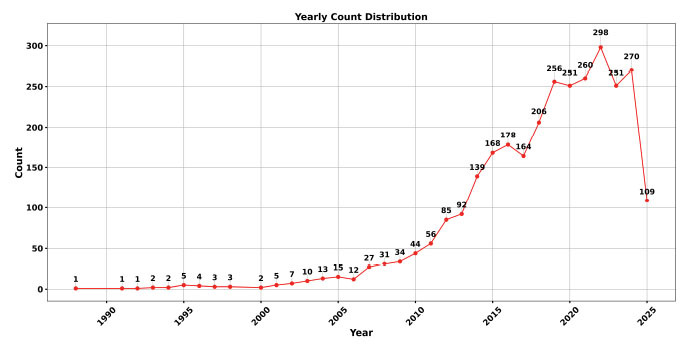
PubMed query used for data collection.

**Fig. (4):**
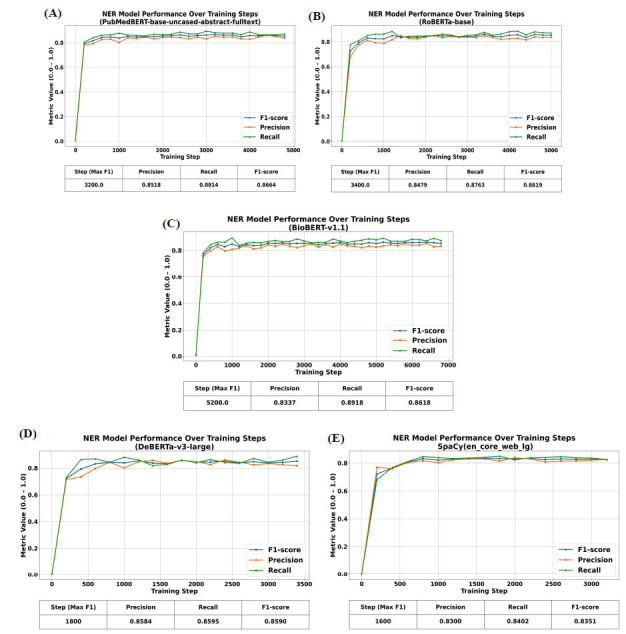
Performance comparison of five NER models over training steps.

**Fig. (5):**
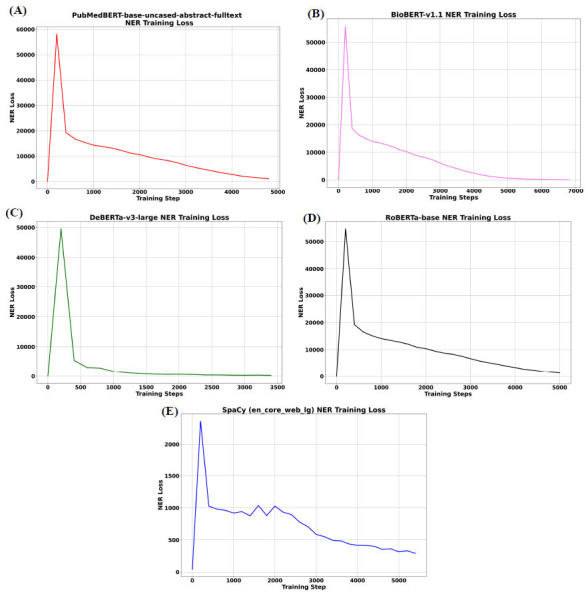
NER training loss.

**Fig. (6):**
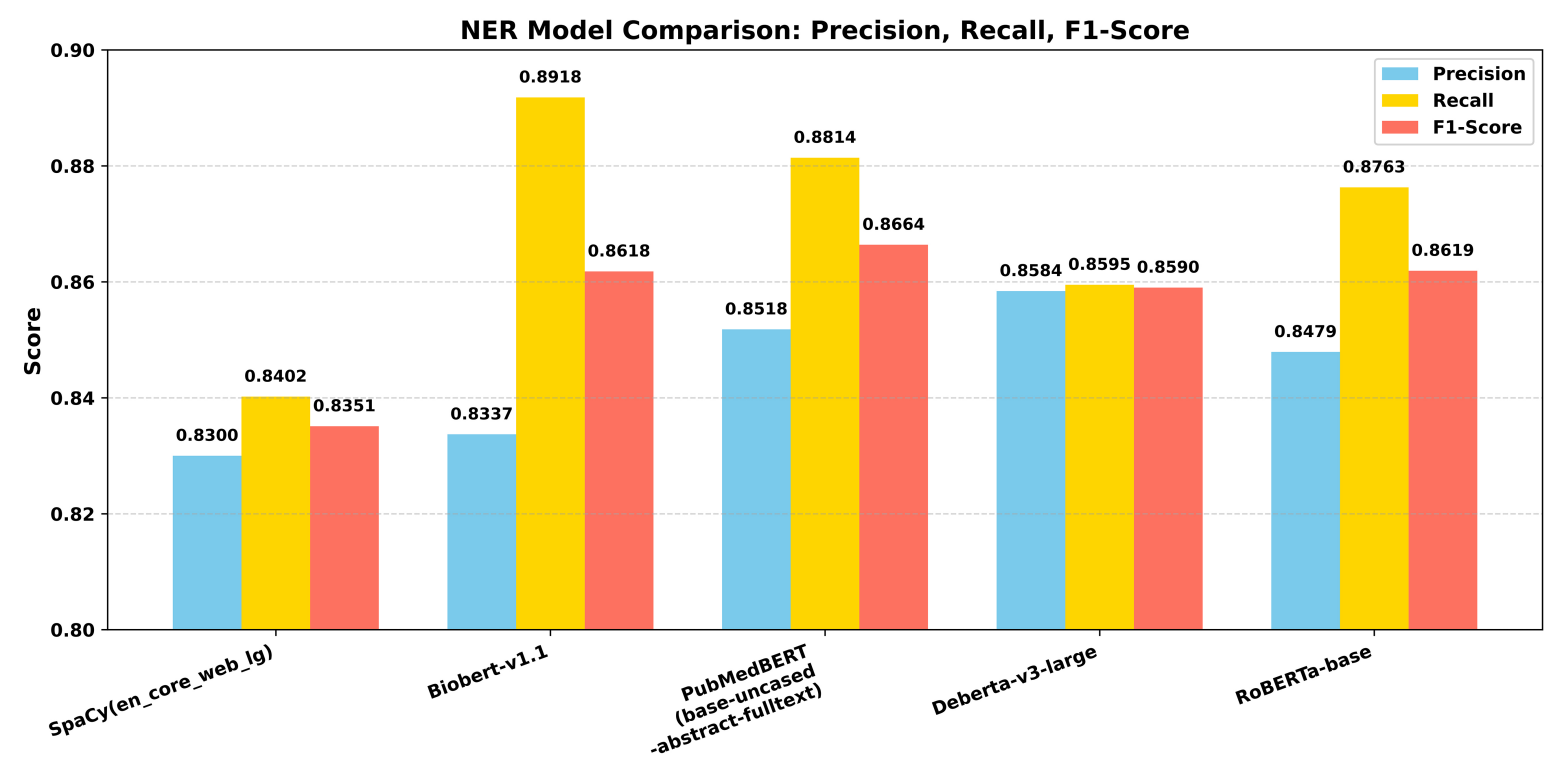
Performance comparison of finetuned models on validation dataset.

**Fig. (7):**
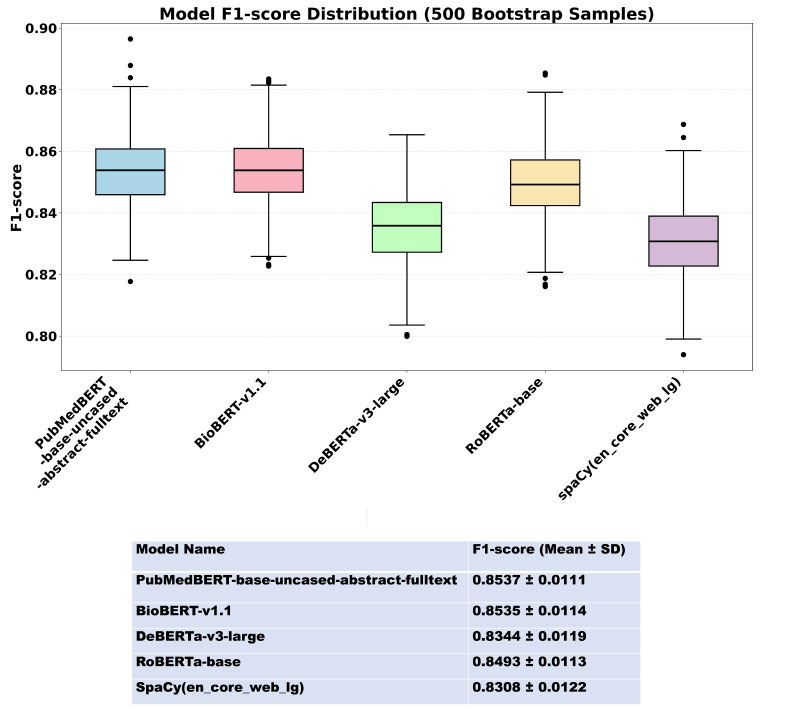
Model F1-score on test dataset.

**Fig. (8):**
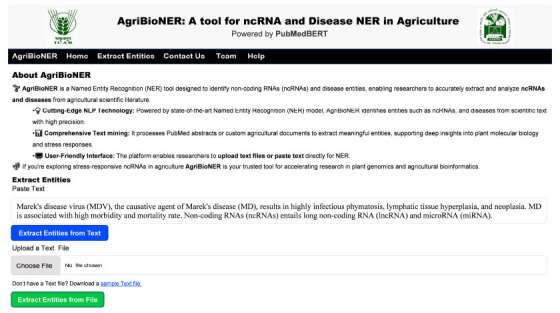
AgriBioNER Web-Interface.

**Fig. (9):**
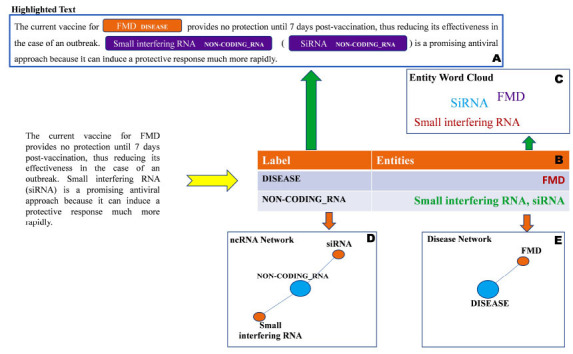
Input and output components of AgriBioNER.

**Table 1:** Entity label distribution across dataset splits.

**Dataset Split**	**Sentences**	**Disease**	**Non-Coding_RNA**
Train	8,624	6,498	7,847
Validation	1,078	829	937
Test	1,078	815	998
Total	10,780	8,142	9,782

**Table 2:** Runtime environment summary.

**Parameter**	**Value**
SpaCy version	3.8.5
Platform	Linux-6.1.123+-x86_64-with-glibc2.35
Python version	3.11.12
Pipeline	en_core_web_trf (3.8.0)

**Table 3:** Model training time.

**Model**	**Training Time**
PubMedBERT-base-uncased-abstract-fulltext	34.01 min
BioBERT-v1.1	52.58 min
DeBERTa-v3-large	59.00 min
RoBERTa-base	56.97 min
SpaCy (en_core_web_lg)	3.87 min

**Table 4:** Entity-wise performance for disease.

**Model**	**Precision (P)**	**Recall (R)**	**F1-score (F1)**
BioBERT-v1.1	0.6875	0.7190	0.7029
PubMedBERT-base-uncased-abstract-fulltext	0.7333	0.7190	0.7261
RoBERTa-base	0.7086	0.6993	0.7039
DeBERTa-v3-large	0.6879	0.7059	0.6968
SpaCy (en_core_web_lg)	0.5686	0.6444	0.6042

**Table 5:** Entity-wise performance for NON-CODING_RNA.

**Model**	**Precision**	**Recall**	**F1**
BioBERT-v1.1	0.861386	0.942724	0.900222
PubMedBERT-base-uncased-abstract-fulltext	0.872279	0.930341	0.900375
RoBERTa-base	0.874453	0.927245	0.900075
DeBERTa-v3-large	0.891304	0.888545	0.889922
SpaCy (en_core_web_lg)	0.873303	0.896285	0.884645

## Data Availability

The authors confirm that the data supporting the findings of this research are available on Zenodo. The Datasets and Training Code can be accessed *via* the following link: https://zenodo.org/records/17814856?token=eyJhbGciOiJIU zUxMiJ9.eyJpZCI6ImUyN2E1NzNjLTgxYmQtNDhkYS1i ZTc5LWEyMWNhMzE4ZGY4NiIsImRhdGEiOnt9LCJyY W5kb20iOiIzODBiYjRjZjM1YTkwMDkwNTY2ZDZhOG FiZWFmNjk0YyJ9.aYZUMcdWq2rsXqVgRJcUL6yNuNR KJ36Nu-_FA7cW9aIOOibMkjTJ006qRcuslGZxPAoNz6O wsuaFo1Iokmfkhw

## LIST OF ABBREVIATIONS

cirRNAs: Circular RNAs
FN: False Negatives (model failed to identify a true entity)
FP: False Positives (model predicted an entity that is not actually an entity)
JSON: JavaScript Object Notation
LLM: Large Language Model
lncRNAs: Long non-coding RNAs
mRNA: Messenger RNA
ncRNA: Non-coding RNA
NER: Named Entity Recognition
NLP: Natural Language Processing
SD: Standard Deviation
sRNAs: Small RNAs
TP: True Positives (model correctly predicted entities)
