# Supplementary material for: AgriBioNER: A Named Entity Recognition Tool for Identification of ncRNA and Diseases in Agricultural Literature
Source: Curr Genomics. 2026 Apr 15;26(7):819–32. doi: 10.2174/0113892029435693260407094051 (PMC13154228; doi:10.2174/0113892029435693260407094051)
Supplement: Supplementary file 1 [file CG-26-7-819_SD1.pdf]

Supplementary Material

AgriBioNER: A Named Entity Recognition Tool for Identification of ncRNA and Diseases in Agricultural Literature

Bhavesh Kumar Choubisa[1], Anu Sharma[1,*], K.K. Chaturvedi[1], Ragini Kushwaha[1], Mohammad Samir Farooqi[1], Dwijesh Chandra Mishra[1], S.B. Lal[1], Sudhir Srivastava[1], Amolkumar U. Solanke[2], Alka Arora[1] and Girish Kumar Jha[1]

[1]ICAR-IASRI, PUSA, New Delhi, 110012, India; [2]ICAR-NIPB, PUSA, New Delhi, 110012, India

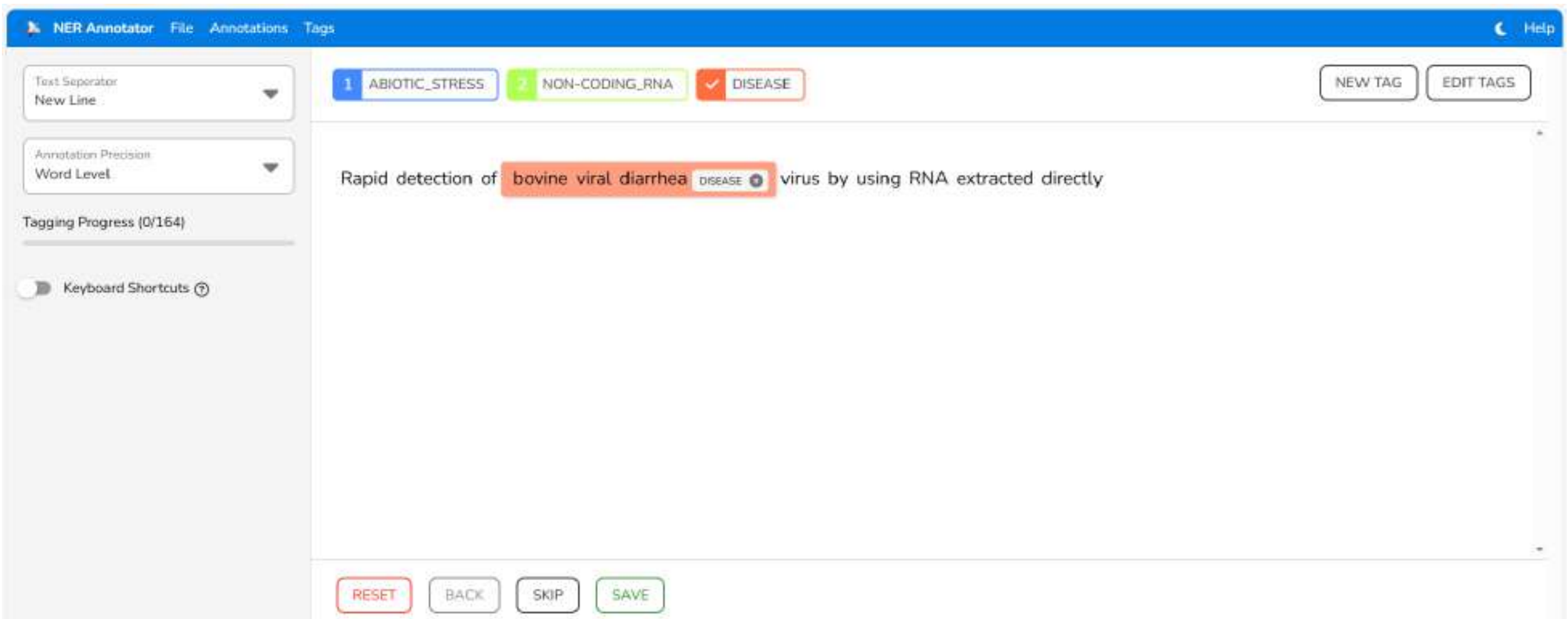

Fig. (S1). Web interface of the NER Annotator. The NER annotation tool built for SpaCy allows users to label text efficiently and export annotations in JSON format. The generated JSON files are directly compatible with SpaCy training pipelines, enabling seamless creation of high-quality training datasets.

Table S1: PubMedBERT-base-uncased-abstract-fulltext Model Hyperparameters.

Hyperparameter	Value
return_dict	True
output_hidden_states	False
output_attentions	False
torchscript	False
torch_dtype	None
use_bfloat16	False
tf_legacy_loss	False
pruned_heads	{}
tie_word_embeddings	True
is_encoder_decoder	False
is_decoder	False
cross_attention_hidden_size	None
add_cross_attention	False
tie_encoder_decoder	False
max_length	20
min_length	0
do_sample	False
early_stopping	False

num_beams	1
num_beam_groups	1
diversity_penalty	0.0
temperature	1.0
top_k	50
top_p	1.0
typical_p	1.0
repetition_penalty	1.0
length_penalty	1.0
no_repeat_ngram_size	0
encoder_no_repeat_ngram_size	0
bad_words_ids	None
num_return_sequences	1
chunk_size_feed_forward	0
output_scores	False
return_dict_in_generate	False
forced_bos_token_id	None
forced_eos_token_id	None
remove_invalid_values	False
exponential_decay_length_penalty	None
suppress_tokens	None
begin_suppress_tokens	None
architectures	['BertForMaskedLM']
finetuning_task	None
id2label	{0: 'LABEL_0', 1: 'LABEL_1'}
label2id	{'LABEL_0': 0, 'LABEL_1': 1}
tokenizer_class	None
prefix	None
bos_token_id	None
pad_token_id	0
eos_token_id	None
sep_token_id	None
decoder_start_token_id	None
task_specific_params	None
problem_type	None
_name_or_path	microsoft/BiomedNLP-PubMedBERT-base-uncased-abstract-fulltext
transformers_version	4.36.2
model_type	bert
vocab_size	30522
hidden_size	768
num_hidden_layers	12
num_attention_heads	12
hidden_act	gelu
intermediate_size	3072
hidden_dropout_prob	0.1
attention_probs_dropout_prob	0.1
max_position_embeddings	512
type_vocab_size	2
initializer_range	0.02
layer_norm_eps	1e-12
position_embedding_type	absolute
use_cache	True
classifier_dropout	None

Table S2: BioBERT-v1.1 Model Hyperparameters.

Hyperparameter	Value
return_dict	True
output_hidden_states	False
output_attentions	False
torchscript	False
torch_dtype	None
use_bfloat16	False
tf_legacy_loss	False
pruned_heads	{}
tie_word_embeddings	True
is_encoder_decoder	False
is_decoder	False
cross_attention_hidden_size	None
add_cross_attention	False
tie_encoder_decoder	False
max_length	20
min_length	0
do_sample	False
early_stopping	False
num_beams	1
num_beam_groups	1
diversity_penalty	0.0
temperature	1.0
top_k	50
top_p	1.0
typical_p	1.0
repetition_penalty	1.0
length_penalty	1.0
no_repeat_ngram_size	0
encoder_no_repeat_ngram_size	0
bad_words_ids	None
num_return_sequences	1
chunk_size_feed_forward	0
output_scores	False
return_dict_in_generate	False
forced_bos_token_id	None
forced_eos_token_id	None
remove_invalid_values	False
exponential_decay_length_penalty	None
suppress_tokens	None
begin_suppress_tokens	None
architectures	None
finetuning_task	None
id2label	{0: 'LABEL_0', 1: 'LABEL_1'}
label2id	{'LABEL_0': 0, 'LABEL_1': 1}
tokenizer_class	None

prefix	None
bos_token_id	None
pad_token_id	0
eos_token_id	None
sep_token_id	None
decoder_start_token_id	None
task_specific_params	None
problem_type	None
_name_or_path	dmis-lab/biobert-base-cased-v1.1
transformers_version	4.36.2
vocab_size	28996
hidden_size	768
num_hidden_layers	12
num_attention_heads	12
hidden_act	gelu
intermediate_size	3072
hidden_dropout_prob	0.1
attention_probs_dropout_prob	0.1
max_position_embeddings	512
type_vocab_size	2
initializer_range	0.02
layer_norm_eps	1e-12
position_embedding_type	absolute
use_cache	True
classifier_dropout	None
model_type	bert

Table S3: DeBERTa-v3-large Model Hyperparameters.

Hyperparameter	Value
return_dict	True
output_hidden_states	False
output_attentions	False
torchscript	False
torch_dtype	None
use_bfloat16	False
tf_legacy_loss	False
pruned_heads	{}
tie_word_embeddings	True
is_encoder_decoder	False
is_decoder	False
cross_attention_hidden_size	None
add_cross_attention	False
tie_encoder_decoder	False
max_length	20
min_length	0
do_sample	False
early_stopping	False

num_beams	1
num_beam_groups	1
diversity_penalty	0.0
temperature	1.0
top_k	50
top_p	1.0
typical_p	1.0
repetition_penalty	1.0
length_penalty	1.0
no_repeat_ngram_size	0
encoder_no_repeat_ngram_size	0
bad_words_ids	None
num_return_sequences	1
chunk_size_feed_forward	0
output_scores	False
return_dict_in_generate	False
forced_bos_token_id	None
forced_eos_token_id	None
remove_invalid_values	False
exponential_decay_length_penalty	None
suppress_tokens	None
begin_suppress_tokens	None
architectures	None
finetuning_task	None
id2label	{0: 'LABEL_0', 1: 'LABEL_1'}
label2id	{'LABEL_0': 0, 'LABEL_1': 1}
tokenizer_class	None
prefix	None
bos_token_id	None
pad_token_id	0
eos_token_id	None
sep_token_id	None
decoder_start_token_id	None
task_specific_params	None
problem_type	None
_name_or_path	microsoft/deberta-v3-large
transformers_version	4.36.2
model_type	deberta-v2
position_buckets	256
norm_rel_ebd	layer_norm
share_att_key	True
hidden_size	1024
num_hidden_layers	24
num_attention_heads	16
intermediate_size	4096
hidden_act	gelu
hidden_dropout_prob	0.1

attention_probs_dropout_prob	0.1
max_position_embeddings	512
type_vocab_size	0
initializer_range	0.02
relative_attention	True
max_relative_positions	-1
position_biased_input	False
pos_att_type	['p2c', 'c2p']
vocab_size	128100
layer_norm_eps	1e-07
pooler_hidden_size	1024
pooler_dropout	0
pooler_hidden_act	gelu

Table S4: RoBERTa-base Model Hyperparameters.

Hyperparameter	Value
return_dict	True
output_hidden_states	False
output_attentions	False
torchscript	False
torch_dtype	None
use_bfloat16	False
tf_legacy_loss	False
pruned_heads	{}
tie_word_embeddings	True
is_encoder_decoder	False
is_decoder	False
cross_attention_hidden_size	None
add_cross_attention	False
tie_encoder_decoder	False
max_length	20
min_length	0
do_sample	False
early_stopping	False
num_beams	1
num_beam_groups	1
diversity_penalty	0.0
temperature	1.0
top_k	50
top_p	1.0
typical_p	1.0
repetition_penalty	1.0
length_penalty	1.0
no_repeat_ngram_size	0
encoder_no_repeat_ngram_size	0
bad_words_ids	None
num_return_sequences	1

chunk_size_feed_forward	0
output_scores	False
return_dict_in_generate	False
forced_bos_token_id	None
forced_eos_token_id	None
remove_invalid_values	False
exponential_decay_length_penalty	None
suppress_tokens	None
begin_suppress_tokens	None
architectures	['RobertaForMaskedLM']
finetuning_task	None
id2label	{0: 'LABEL_0', 1: 'LABEL_1'}
label2id	{'LABEL_0': 0, 'LABEL_1': 1}
tokenizer_class	None
prefix	None
bos_token_id	0
pad_token_id	1
eos_token_id	2
sep_token_id	None
decoder_start_token_id	None
task_specific_params	None
problem_type	None
_name_or_path	roberta-base
transformers_version	4.36.2
model_type	roberta
vocab_size	50265
hidden_size	768
num_hidden_layers	12
num_attention_heads	12
hidden_act	gelu
intermediate_size	3072
hidden_dropout_prob	0.1
attention_probs_dropout_prob	0.1
max_position_embeddings	514
type_vocab_size	1
initializer_range	0.02
layer_norm_eps	1e-05
position_embedding_type	absolute
use_cache	True
classifier_dropout	None

Table S5: SpaCy (en_core_web_lg) Model Hyperparameters.

Parameter	Value
Pipeline components	['tok2vec', 'tagger', 'parser', 'attribute_ruler', 'lemmatizer', 'ner']
Vocabulary size	764
Vector length	300

Number of vectors	342918
Tokenizer rules	1347

Table S6: Training Performance PubMedBERT-base-uncased-abstract-fulltext.

Step	Precision	Recall	F1-score
0.0	0.0036	0.0258	0.0063
200.0	0.782	0.8041	0.7929
400.0	0.7922	0.8402	0.8155
600.0	0.8257	0.8608	0.8429
800.0	0.8284	0.8647	0.8462
1000.0	0.8009	0.8763	0.8369
1200.0	0.8385	0.8634	0.8508
1400.0	0.8346	0.8582	0.8463
1600.0	0.8446	0.8544	0.8495
1800.0	0.8313	0.8698	0.8501
2000.0	0.8421	0.866	0.8539
2200.0	0.8436	0.8686	0.8559
2400.0	0.8411	0.8866	0.8632
2600.0	0.8325	0.8711	0.8514
2800.0	0.8439	0.8711	0.8573
3000.0	0.8319	0.893	0.8614
**3200.0**	**0.8518**	**0.8814**	**0.8664**
3400.0	0.8437	0.8763	0.8597
3600.0	0.8472	0.8789	0.8627
3800.0	0.8315	0.8647	0.8478
4000.0	0.8281	0.8879	0.857
4200.0	0.8451	0.8647	0.8548
4400.0	0.8601	0.8634	0.8617
4600.0	0.8494	0.8647	0.857
4800.0	0.8366	0.8711	0.8535

Table S7: Training Performance RoBERTa-base.

Step	Precision	Recall	F1-score
0.0	0.004	0.0052	0.004
200.0	0.6809	0.7784	0.7264
400.0	0.7753	0.8093	0.7919
600.0	0.8128	0.8505	0.8312
800.0	0.7927	0.8621	0.8259
1000.0	0.7889	0.8621	0.8239
1200.0	0.8176	0.884	0.8495
1400.0	0.8502	0.8338	0.8419
1600.0	0.8283	0.8454	0.8367
1800.0	0.8254	0.8466	0.8359
2000.0	0.8372	0.8479	0.8425
2200.0	0.8503	0.8492	0.8498
2400.0	0.8323	0.8634	0.8476

2600.0	0.8424	0.8544	0.8484
2800.0	0.837	0.8402	0.8386
3000.0	0.8367	0.8518	0.8442
3200.0	0.8344	0.857	0.8455
**3400.0**	**0.8479**	**0.8763**	**0.8619**
3600.0	0.8361	0.8544	0.8451
3800.0	0.8201	0.8634	0.8412
4000.0	0.8251	0.8814	0.8523
4200.0	0.8297	0.8853	0.8566
4400.0	0.815	0.857	0.8354
4600.0	0.8391	0.8802	0.8591
4800.0	0.835	0.8737	0.8539
5000.0	0.8366	0.8711	0.8535

Table S8: Training Performance BioBERT-v1.1

**Step**	**Precision**	**Recall**	**F1-score**
0.0	0.0111	0.0103	0.0107
200.0	0.7537	0.7809	0.7671
400.0	0.7966	0.8428	0.8190
600.0	0.828	0.8621	0.8447
800.0	0.7964	0.8621	0.8280
1000.0	0.807	0.8943	0.8484
1200.0	0.8186	0.8376	0.8280
1400.0	0.8361	0.8544	0.8451
1600.0	0.8107	0.8608	0.8350
1800.0	0.8192	0.8582	0.8383
2000.0	0.8412	0.8673	0.8541
2200.0	0.8301	0.875	0.8519
2400.0	0.8474	0.866	0.8566
2600.0	0.8319	0.8673	0.8492
2800.0	0.8208	0.8853	0.8518
3000.0	0.8348	0.8724	0.8532
3200.0	0.8463	0.8582	0.8522
3400.0	0.8247	0.8608	0.8424
3600.0	0.8424	0.8608	0.8515
3800.0	0.8265	0.884	0.8543
4000.0	0.8471	0.8711	0.8590
4200.0	0.8338	0.8595	0.8464
4400.0	0.8311	0.8686	0.8494
4600.0	0.8203	0.8763	0.8474
4800.0	0.8329	0.8866	0.8589
5000.0	0.8247	0.8789	0.8509
**5200.0**	**0.8337**	**0.8918**	**0.8618**
5400.0	0.8406	0.8698	0.8550
5600.0	0.8342	0.8686	0.8510

5800.0	0.851	0.8686	0.8597
6000.0	0.8364	0.8827	0.8589
6200.0	0.8382	0.8814	0.8593
6400.0	0.8525	0.8711	0.8617
6600.0	0.8275	0.8905	0.8579
6800.0	0.8319	0.8737	0.8523

Table S9: Training Performance DeBERTa-v3-large.

Step	Precision	Recall	F1-score
0.0	0.0028	0.0052	0.0037
200.0	0.7159	0.7307	0.7232
400.0	0.733	0.8634	0.7929
600.0	0.7969	0.8698	0.8318
800.0	0.8441	0.8441	0.8441
1000.0	0.8019	0.8814	0.8398
1200.0	0.8479	0.8621	0.8550
1400.0	0.8581	0.8183	0.8377
1600.0	0.8362	0.8286	0.8324
**1800.0**	**0.8584**	**0.8595**	**0.8590**
2000.0	0.8481	0.8415	0.8448
2200.0	0.8264	0.8647	0.8451
2400.0	0.8617	0.8428	0.8521
2600.0	0.8451	0.8363	0.8407
2800.0	0.8246	0.8724	0.8478
3000.0	0.8344	0.8441	0.8392
3200.0	0.8257	0.8608	0.8429
3400.0	0.8175	0.8892	0.8519

Table S10: Training Performance SpaCy (en_core_web_lg).

Step	Precision	Recall	F1-score
0.0	0.0	0.0	0.0
200.0	0.7683	0.6798	0.7213
400.0	0.7611	0.7684	0.7647
600.0	0.8032	0.8105	0.8068
800.0	0.8166	0.8467	0.8314
1000.0	0.8032	0.8396	0.8210
1200.0	0.8241	0.8331	0.8286
1400.0	0.8295	0.837	0.8332
**1600.0**	**0.8300**	**0.8402**	**0.8351**
1800.0	0.8142	0.8506	0.8320
2000.0	0.8391	0.8228	0.8308
2200.0	0.8298	0.8389	0.8344
2400.0	0.8073	0.8402	0.8235
2600.0	0.8141	0.8467	0.8301
2800.0	0.8151	0.8383	0.8265

3000.0	0.8215	0.8364	0.8288
3200.0	0.8248	0.8254	0.8251

Table S11: PubMedBERT-base-uncased-abstract-fulltext NER Loss.

Training Step	NER Loss
0	53.94
200	58154.32
400	19301.94
600	16724.55
800	15430.01
1000	14321.46
1200	13770.48
1400	13148.67
1600	12268.19
1800	11201.23
2000	10596.66
2200	9601.79
2400	8847.89
2600	8278.0
2800	7483.39
3000	6395.39
3200	5527.51
3400	4790.03
3600	4083.11
3800	3367.92
4000	2764.7
4200	2156.77
4400	1741.57
4600	1387.13
4800	1060.68

Table S12: BioBERT-v1.1 NER Loss.

Training Step	NER Loss
0	47.71
200	55815.39
400	18643.25
600	16282.49
800	15040.81
1000	13979.36
1200	13461.77
1400	12842.13
1600	12034.88
1800	10914.9
2000	10322.75
2200	9251.19
2400	8555.63
2600	7963.49

2800	7160.09
3000	6090.79
3200	5183.63
3400	4424.75
3600	3702.44
3800	2982.29
4000	2410.7
4200	1864.03
4400	1416.77
4600	1091.68
4800	828.79
5000	585.27
5200	450.28
5400	365.0
5600	232.19
5800	191.51
6000	188.86
6200	108.54
6400	104.06
6600	78.59
6800	65.02

Table S13: DeBERTa-v3-large NER Loss.

Training Step	NER Loss
0	65.02
200	49575.77
400	5420.67
600	3055.47
800	2851.6
1000	1730.69
1200	1194.41
1400	957.89
1600	740.17
1800	618.62
2000	664.51
2200	583.04
2400	421.3
2600	421.67
2800	337.86
3000	310.3
3200	352.05
3400	271.79

Table S14: RoBERTa-base NER Loss.

Training Step	NER Loss
0	45.24

200	54685.57
400	19199.57
600	16501.17
800	15111.75
1000	14073.22
1200	13469.01
1400	12818.85
1600	12047.35
1800	10900.46
2000	10388.93
2200	9463.17
2400	8772.23
2600	8325.04
2800	7593.6
3000	6670.79
3200	5829.77
3400	5195.82
3600	4616.0
3800	3946.07
4000	3390.81
4200	2773.74
4400	2401.49
4600	1997.17
4800	1633.79
5000	1308.38

Table S15: SpaCy (en_core_web_lg) NER Loss.

Training Step	NER Loss
0	45.7
200	2357.75
400	1026.13
600	980.2
800	961.81
1000	920.13
1200	942.55
1400	875.7
1600	1038.58
1800	879.9
2000	1029.64
2200	933.0
2400	895.47
2600	777.54
2800	706.4
3000	587.51
3200	551.31
3400	495.23
3600	486.28

3800	437.74
4000	417.83
4200	414.63
4400	402.67
4600	354.95
4800	363.93
5000	316.61
5200	333.1
5400	292.77

Table S16: Model Performance (Mean ± SD) on Test Data.

Rank	Model	Precision (Mean ± SD)	Recall (Mean ± SD)	F1-score (Mean ± SD)
1	PubMedBERT-base-uncased-abstract-fulltext	0.8351 ± 0.0139	0.8732 ± 0.0117	0.8537 ± 0.0111
2	BioBERT-v1.1	0.8214 ± 0.0146	0.8882 ± 0.0112	0.8535 ± 0.0114
3	DeBERTa-v3-large	0.8373 ± 0.0144	0.8317 ± 0.0140	0.8344 ± 0.0119
4	RoBERTa-base	0.8303 ± 0.0140	0.8693 ± 0.0122	0.8493 ± 0.0113
5	SpaCy (en_core_web_lg)	0.8389 ± 0.0148	0.8230 ± 0.0141	0.8308 ± 0.0122

Table S17: McNemar and Bootstrap Significance Test Results.

Model 1	Model 2	McNemar_p	Bootstrap_p
BioBERT-v1.1	SpaCy (en_core_web_lg)	0.001049607	0.49
PubMedBERT-base-uncased-abstract-fulltext	BioBERT-v1.1	0.897421827	0.906
PubMedBERT-base-uncased-abstract-fulltext	DeBERTa-v3-large	0.050250895	0.508
PubMedBERT-base-uncased-abstract-fulltext	RoBERTa-base	0.614655016	0.712
PubMedBERT-base-uncased-abstract-fulltext	SpaCy (en_core_web_lg)	0.002394629	0.516
DeBERTa-v3-large	BioBERT-v1.1	0.029456362	0.486
DeBERTa-v3-large	RoBERTa-base	0.155656464	0.486
DeBERTa-v3-large	SpaCy (en_core_web_lg)	0.307089664	0.71
RoBERTa-base	BioBERT-v1.1	0.476687793	0.68
RoBERTa-base	SpaCy (en_core_web_lg)	0.016745021	0.496

Table S18: Quantitative Error Analysis of NER Models.

Model	False Positive	False Negative	Entity Error %
BioBERT-v1.1	153	89	25.61
PubMedBERT-base-uncased-abstract-fulltext	138	101	25.7
RoBERTa-base	141	104	26.26

DeBERTa-v3-large	128	133	28.37
SpaCy (en_core_web_lg)	125	140	28.9

Table S19: Common Qualitative Errors of Models.

Text	True Entities	Predicted Entities	False Positive	False Negative
Rapid detection of bovine viral diarrhea virus by using RNA extracted directly	[[26, 40, 'DISEASE']]	[(32, 40, 'DISEASE')]	1	1
These findings provide a comprehensive view of potentially functional lncRNAs, corresponding target genes and related lncRNA-miRNA pairs, which will greatly increase our knowledge of the mechanism underlying susceptibility and resistance to PM in melon.	[[71, 78, 'NON-CODING_RNA']]	[(71, 78, 'NON-CODING_RNA'), (244, 246, 'DISEASE')]	1	0
Swine hepatitis E (swine HE) is a new type of zoonotic infectious disease caused	[[6, 15, 'DISEASE']]	[(6, 17, 'DISEASE'), (25, 27, 'DISEASE')]	2	1
Bovine mastitis is an inflammation-driven disease of the bovine mammary gland	[[7, 15, 'DISEASE']]	[(0, 15, 'DISEASE')]	1	1
Further, co-transfection of miR-424-5p inhibitors and siRNA-IRAK2 revealed that negative regulation of miR-424-5p on LPS-induced inflammatory response in BEECs was mediated by IRAK2.Mutually, miR-424-5p pharmacologic stabilization represents an entirely unique medical aid for cow endometritis and other inflammation-related diseases.	[[28, 38, 'NON-CODING_RNA'], [54, 65, 'NON-CODING_RNA'], [104, 114, 'NON-CODING_RNA'], [194, 218, 'NON-CODING_RNA'], [284, 296, 'DISEASE']]	[(28, 38, 'NON-CODING_RNA'), (104, 114, 'NON-CODING_RNA'), (194, 204, 'NON-CODING_RNA'), (280, 296, 'DISEASE')]	2	3
miRanda, RNA22, Tapirhybrid, and psRNA. The goal is to deploy the predicted	[[33, 39, 'NON-CODING_RNA']]	[(0, 7, 'NON-CODING_RNA'), (9, 14, 'NON-CODING_RNA'),	3	0

		(16, 27, 'NON-CODING_RNA'), (33, 39, 'NON-CODING_RNA')]		
miRNA were profiled, including 331 known and 55 novel miRNA sequences, of which	[[0, 5, 'NON-CODING_RNA']]	[(0, 5, 'NON-CODING_RNA'), (54, 59, 'NON-CODING_RNA')]	1	0

Table S20: Baseline Model Performance on Domain-specific Test Dataset.

Baseline Model	Precision	Recall	F1-score	False Positive	False Negative	Entity Error %
**en_ner_bc5cdr_md**	0.1393	0.0429	0.0656	210	758	96.61
**en_ner_bionlp13cg_md**	0.0	0.0	0.0	742	792	100.0
**en_ner_jnlpba_md**	0.0	0.0	0.0	611	792	100.0

Table S21: Latency Metrics PubMedBERT-base-uncased-abstract-fulltext NER Model.

Metric	Value
latency_max_sec	3.5620110034942627
words_per_sec_max	202.2674011558095
sentences_per_sec_max	10.155685413680812
abstracts_per_sec_max	0.8463071178067343

Table S22: Batch Processing Times PubMedBERT-base-uncased-abstract-fulltext NER Model.

Metric	Value
batch_1_max_time_sec	1.774122953414917
batch_5_max_time_sec	9.12311577796936
batch_10_max_time_sec	17.041579961776733
batch_20_max_time_sec	20.597078561782837
batch_50_max_time_sec	22.013769149780273
batch_100_max_time_sec	20.56523060798645

Table S23: Stress Test PubMedBERT-base-uncased-abstract-fulltext NER Model.

Metric	Value
stress_long_doc_chars	167050
stress_time_sec	222.1257734298706
